# Qualitative Examination of the Role and Influence of Mothers-in-Law on Young Married Couples’ Family Planning in Rural Maharashtra, India

**DOI:** 10.9745/GHSP-D-22-00050

**Published:** 2022-10-31

**Authors:** Anvita Dixit, Mohan Ghule, Namratha Rao, Madhusudana Battala, Shahina Begum, Nicole E. Johns, Sarah Averbach, Anita Raj

**Affiliations:** a Center on Gender Equity and Health, Division of Infectious Diseases and Global Public Health, School of Medicine, University of California San Diego, San Diego, CA, USA.; b Faculty of Health Sciences, University of Ottawa, Ottawa, Canada.; c Population Council, New Delhi, India.; d ICMR-National Institute for Research in Reproductive Health, Mumbai, India.; e Department of Obstetrics, Gynecology, and Reproductive Sciences, School of Medicine, University of California San Diego, San Diego, CA, USA.; f Department of Education Studies, Division of Social Sciences, University of California San Diego, San Diego, CA, USA.

## Abstract

Unmet need for family planning (FP) continues to be high in India, especially among young and newly married women. Mothers-in-law (MILs) often exert pressure on couples for fertility and control decision making and behaviors around fertility and FP, yet there is a paucity of literature to understand their perspectives. Ten focus group discussions (FGDs) were carried out with MILs of young married women (aged 18–29 years) participating in a couple-focused FP intervention as a part of a cluster-randomized intervention evaluation trial (the CHARM2 study) in rural Maharashtra, India. FGDs included questions on their roles, attitudes, and decision making around fertility and FP. Audio-recorded data were translated/transcribed into English and analyzed for key themes using a deductive coding method. MILs reported having social norms of early fertility and son preference. They understood that family size norms are lower among daughters-in-law and that spacing can be beneficial but were not supportive of short-term contraceptives, especially before the first child. They preferred female sterilization, opposed abortion, had apprehensions around side effects from contraceptive use, and had misconceptions about the intrauterine device, with particular concerns around its coercive insertion. MILs mostly believed that decision making should be done jointly by a husband and wife, but that as elders, they should be consulted and involved in the decision-making process. These findings highlight the need for engagement of MILs for FP promotion in rural India and the potential utility of social norms interventions.

## Abstract (precis)

Mothers-in-law (MILs) in India hold influential norms that can compromise the reproductive autonomy of their daughters-in-law. Family planning interventions should address MILs’ attitudes and involvement in reproductive decision making.

## Teaser

**Key Findings:**

- Mothers-in-law (MILs) had strong preferences for early births in marriage and preferred female sterilization over other modern forms of short-term contraception such as pills and intrauterine devices.
- MILs held traditional gender role attitudes and expected daughters-in-law to adhere to these. While they supported birth spacing and joint couple decision making, they also wanted to be included in family planning (FP) decision making.

**Key Implications:**

- Given their potential influence on couples’ FP behaviors, MILs should be engaged in FP promotion in rural India.
- National stakeholders can potentially include components to address MILs’ gender and fertility norms in FP interventions.

## INTRODUCTION

Approximately 1 in 8 women of reproductive age (15–49 years) report unmet need for family planning (FP) in India. For 20 years, there has not been a significant decline in the prevalence of unmet need (with a slight decrease from 16% in 1998–1999 to 13.9% in 2005–2006 and 12.9% in 2015–2016), despite an increase in the availability of contraceptive methods.^1^^,^^2^ Nationally and in Maharashtra, 8% of girls aged 15–19 years have already begun childbearing.^1^ Young adult women in India are among those with the highest unmet need, with approximately 20% of women aged 18–29 years reporting unmet need.^1^ Evidence suggests that many Indian women early in marriage face high fertility pressure from extended family due to prevalent social norms and expectations related to early birth as an indicator of a healthy marriage and son preference due to beliefs that sons, rather than daughters, can provide longer-term security for families, particularly those more socially or economically vulnerable.^3^^–^^5^ Mothers-in-law (MILs), in particular, have been identified as the messengers of fertility pressures for young couples in India.^5^^–^^10^ Further, research from India on reproductive coercion (i.e., the coercion of women’s reproductive control by their husband and/or other household and family members) also demonstrates that in-laws, more than husbands, perpetrate this type of abusive fertility control.^11^ Reproductive coercion is associated with intimate partner violence and predicts reproductive outcomes such as contraceptive use and unintended pregnancy.^12^^,^^13^

Research from India on reproductive coercion demonstrates that in-laws, more than husbands, perpetrate this type of abusive fertility control.

Reproductive coercion has a direct bearing on realizing reproductive justice for women in this context. Given the important role of in-laws in reproductive health in India, in-law involvement has been included in assessments of reproductive coercion.^14^^–^^16^ Ensuring reproductive justice includes the right to maintain bodily autonomy and reproductive decision making.^17^

Taken together, these studies demonstrate the important role MILs have on fertility practices and reproductive decision making among young married couples, resulting in recommendations for MILs to be engaged in FP intervention efforts.^18^^,^^19^ The national FP program in 7 states has also added meetings for MILs and daughters-in-law (DILs) called Saas Bahu Sammelans to encourage their communication on reproductive health matters (not yet implemented in Maharashtra).^20^ However, data on perspectives of the MILs themselves on FP use in the household are lacking, which is important to guide their inclusion in intervention efforts and their effective inclusion in FP programs.^21^ This study contributes to filling this evidence gap with a qualitative analysis of focus group data from MILs of married women aged 18–29 years in rural Maharashtra, India.

## METHODS

### Study Design and Sampling

The study was conducted in Junnar taluka (taluka: geographic subdistrict area) located in the Pune district of Maharashtra, India, as part of a larger FP intervention trial, the Counseling Husbands and wives to Achieve Reproductive Health and Marital equity (CHARM2) study. In rural Pune, female illiteracy is 27%, and the child sex ratio is 833 girls per 1,000 boys (indicative of son preference/missing girls).^22^ Only 25% of nonsterilized women of childbearing age use modern contraception.^23^ CHARM2 is a 2-armed cluster randomized control trial (intervention and control conditions) with couples including wives aged 18–29 years and their husbands. It uses a gender-synchronized, gender-transformative counseling intervention to improve contraceptive use as well as reduce unintended pregnancy and marital sexual violence. Details on the larger study are described elsewhere.^24^ After intervention delivery, research staff identified and recruited a sample of MILs among the study participants, randomly selected from each of our 10 geographic intervention clusters, to participate in focus groups. Given that 82% of the intervention couples (80% in the entire study sample) lived in the same household with the MIL, it was important to understand their role and influence. MILs were recruited only from the intervention clusters to study MILs’ opinions about family dynamics between husbands’ parents and young couples, issues related to family and FP, and their opinion on the CHARM2 program. We employed a convenience sampling method, wherein the recruitment team approached MILs through program participants until they had recruited 8–12 participants per FGD.

Female participants were given the option to invite their MILs for recruitment to allow them choice in whether to engage their MIL in the study. Only MILs of those married couples who agreed to our outreach were approached for recruitment into the FGDs to ensure CHARM2 participant privacy and confidentiality and prevent any conflicts between the MILs and couples introduced by their participation in our study. One focus group discussion (FGD) with 8–10 MILs per experimental arm cluster was planned based on the size of the field and our previous experiences with FGDs^25^ as well as for geographic feasibility matching the 10 clusters. The 10 FGDs were carried out in the 10 clusters to prevent any recruitment biases with other clusters and so that MILs did not have to travel long distances for the FGD.

We collected data from MILs through FGDs at the same time as recruitment and baseline survey data collection for the CHARM2 intervention study. This helped ensure that MILs were recruited only from families where couples gave their consent for us to approach the MIL for the FGDs and enabled judicious use of the research team’s effort.

Staff conducted a focus group in each geographic cluster (i.e., 10 focus groups), with 6–11 participants per group, resulting in a total sample of N=86. The selected sample was from a similar socioeconomic background to each other (average CHARM2 intervention arm household monthly income was 23315 Indian rupees [approx. US$300] and 25% holding below poverty line status). MILs were diverse in age, age at marriage, and education (Table). The current age varied from 40 to 75 years with a majority aged 46 to 65 years. Only 3 MILs reported being older than 65 years. Age at marriage ranged from 7 to 23 years, with only 2 reporting being age 7 years at marriage. The majority married between ages 11 and 18 years. The level of education ranged from 0 or no schooling up to the 10^th^ standard, and 19 of them reported 0 years of schooling.

**TABLE.** Sociodemographic Characteristics of Mothers-in-Law Study Participants in Rural Maharashtra, India

**Characteristic**	**N=83** ^a^	**Mean (SD)**
Age range, years	40–75	54.64 (7.46)
Education range, standard	0–10	4.46 (3.32)
Marriage age range, years	7–23	16.46 (2.74)
Number of children	1–7	3.15 (1.24)
Current family living situation, % Joint^b^	83.33	
Nuclear	16.67	

Abbreviation: SD, standard deviation.

^a^ Data are unspecified from 3 mothers-in-law: 2 refused and 1 missing from a focus group with 6 participants.

^b^ Joint family refers to a household where the mother-in-law lives with the son and daughter-in-law.

### Procedure

From August 2019 to February 2020, trained female research staff holding Master’s degrees or higher carried out semistructured FGDs in Marathi that lasted approximately 45 minutes. The semistructured FGD guide assessed family dynamics between husbands’ parents and young couples, in-law engagement in FP awareness or decision making, and in-law perceptions of contraceptives (Supplement 1 includes the focus group guide). The research team encouraged all participants to speak by repeating the questions to elicit more participant responses. After each FGD, the research team made notes on any observed participant dynamics and nonverbal communication.

Before conducting the focus groups, MILs gave their informed written consent. Staff audio-recorded focus groups for transcription/translation and took notes during the group. After the focus group, staff reviewed all notes and audiotapes for quality and de-identification of data. We then transcribed/translated audiotapes into English for analysis.

### Ethical Approval

The institutional review boards of ICMR-National Institute for Research in Reproductive Health in India, Population Council, and the University of California San Diego approved all study procedures.

### Data Analysis

Focus group transcripts were reviewed iteratively. Our research team carried out content and thematic analysis using deductive coding by developing codes based on key areas in the focus group guides and review of the first 2 focus groups. The coders identified new codes iteratively in the coding process, with the final list of codes reviewed and approved by the full scientific study team. Frameworks were developed by coders based on key themes emerging from the FGDs. PhD-level team members conducted analyses using Atlast.ti software. Themes broadly focused on: (1) MIL’s role in family life, (2) DIL’s expected role, (3) norms and attitudes toward FP, and (4) FP decision making.

## RESULTS

### MIL’s Role in Family Life

MILs perceived their role as a parental figure and elder guide for the DIL, a parent and protector of their son, and a caregiver for their grandchildren (Supplement 2). MILs said their role is to guide their DILs about household chores and help them do things correctly if they make mistakes.

MILs perceived their role as a parental figure and elder guide for the DIL, a parent and protector of their son, and a caregiver for their grandchildren.

*We have to guide them …We tell them if they do any mistake …We tell them about household chores …Sometimes the method of doing work is different. We have to do that like our own methods. Sometimes they ask us by themselves. We tell them how to make the vegetables and what we have to put into that. We tell them these things. —*Two MILs, age 48 years, 10^th^ standard education and age 58 years, 7^th^ standard education

Additionally, some MILs reported that their DILs have increased education and awareness levels leading to increased decision-making autonomy compared to when the MILs got married.

*We were minor [age 18 years] at the time we got married. Now these girls get married after they are adult, so they have knowledge of everything. If we tell them anything, they get angry. —*MIL, age 58 years, 7^th^ standard education

*So telling them anything is wrong. We never brought illiterate girls [home after marriage]. They are well educated. —*MIL, age 58 years, 7^th^ standard education

Some MILs also reported expecting some resistance from their DILs to carrying out their perceived role of taking care of grandchildren and ensuring a happy marital life for their son and DIL, perhaps due to increased autonomy among DILs (Supplement 2).

*But if she [DIL] will allow children to come to us then only we can take care of them. She has to understand that grandmothers have some responsibility. She should know this. —*MIL, age 59 years, 2^nd^ standard education

### DIL’s Expected Role

MILs described their expectations from DILs and consistently reported that the DIL should perform activities in line with traditional gender roles for women, including cooking, looking after children, washing dishes and clothes, and caring for the in-laws (Supplement 2).

*She has to cook food twice a day. She has to wash clothes and utensils. She has to take care of grandchildren. She has to check on the in-laws and we expect nothing more than that. —*MIL, age 45 years, 6^th^ standard education

The intergenerational tension between MILs and DILs was highlighted—MILs expected DILs to carry out roles similar to what they did when they got married.

*Our only expectation is they should do the things as we have done (e.g., domestic labor, care for family including in-laws), nothing different than that. —*MIL, age 58 years, 8^th^ standard education

Some MILs were supportive of DILs pursuing further education, but a few also expected their DILs to participate in income generation along with the responsibility of domestic work.

*They should manage their time for family also. They should take care of their children’s future. Whatever we have done for our children, they should do that for their children. —*MIL, age 45 years, 7^th^ standard education

*We never expect everything from daughter-in-law but she should manage her time for her family and her children, we never expect that she should earn more money but she should be careful all the time. —*Two MILs, age 45 years, 7^th^ standard education and age 60 years, 4^th^ standard education

### Norms and Attitudes Toward FP

#### Fertility Norms and Attitudes

MILs believed that couples should have children immediately or soon after marriage, which is also the broader societal expectation (Supplement 2).

*They should have children immediately (after marriage). They should have a first child in 2 to 3 years of their marriage, whether it is a son or daughter. —*MIL, age 58 years, 8^th^ standard education

*After completing 1 year of their marriage everybody expects a child from them.* —MIL, age 68 years, no education

If the DIL delays childbearing, MILs expressed concern for DILs’ health given her older age at conception, describing it as harmful to have a child so late. MILs also expressed concern about being able to help with childcare as they age when conception is delayed. MILs preferred that their DILs not use contraception before having their first child, expressing concern about the effects of contraception on fertility.

MILs preferred that their DILs not use contraception before having their first child, expressing concern about effects of contraception on fertility.

*Yes, if they take contraceptive pills immediately after marriage they get problems.* —MIL, age 40 years, no education

*They can use it after having a child but they should not use it before having a child.* —MIL, age 50 years, 9^th^ standard education

*If they use anything [a contraceptive method] they can’t conceive when they want to have the baby. —*MIL, age 55 years, 10^th^ standard education

Son preference was common among MILs, due to the responsibility of marrying daughters, financial inheritance of land through sons, and norms of living with sons in their old age. In India, paying for the wedding, gifts, or dowry to the groom’s family is considered the bride’s parents’ responsibility, which is often a source of worry and financial burden for them.

*My older son has 2 daughters and younger son has 1 daughter, so I tell them that we should have a son. So my younger son tells me that he will live happily after his daughter’s marriage. —*MIL, age 58 years, 8^th^ standard education

MILs expressed how the notion of son preference is more clandestine but also how it remains a shared attitude by many.

*Now we say that we don’t discriminate, but that is not true, everybody wants a son. —*MIL, age 59 years, 2^nd^ standard education

A few MILs reported that there is no son preference over daughters or discrimination against girls.

In general, more boys than girls attend school in India because of gender social norms. MILs shared that although some girls are well educated in their area, there are differences how education is prioritized for girls and boys.

When asked about ideal family size, MILs expressed a desire for more children, particularly sons, than their DILs but acknowledged that the couples may desire a lower family size.

*We want 10 grandchildren (laughs) but they are saying that they want only one. —*MIL, age 55 years, no education

*So we never advice them anything. They will do whatever they want to do. —*MIL, age 45 years, 7^th^ standard education

#### Contraceptive Norms and Attitudes

Supportive attitudes toward healthy birth spacing between children were noted. MILs reported a different number of ideal years of spacing between children, but these attitudes coexist with son preference norms (Supplement 2).

*Yes, we feel that they should keep space between 2 children. We think that if they have daughter, they should have son after 4 to 5 years. They should have first baby after 2 years of their marriage. —*MIL, age 60 years, 5^th^ standard education

While MILs may be supportive of healthy practices such as birth spacing, they still hold on to the traditional son preference norms, which are a barrier to contraceptive use and may be difficult to change.

A lack of support for contraception use was predominant among MILs, especially for any short-term methods.

A lack of support for contraception use was predominant among MILs, especially for any short-term methods, such as pills and intrauterine devices (IUDs), due to concerns about side effects and the desire for more children.

*No, taking contraceptive pills is not right. Yes, it is not right, we want a child, however it is. —*MIL, age 40 years, no education

*They can do operation immediately after that. —*MIL, age 59 years, 2^nd^ standard education

Female sterilization continues to be the most preferred method to be used after the desired family size is achieved, and MILs were not supportive of male sterilization due to their belief that men have to perform work that is more physically demanding than that done by women. MILs also explained that men’s work brings in a higher income than women’s work, so it is a higher risk for men to have an operation. This highlights how male vasectomy, in the context of India, is disregarded due to poor knowledge and several misconceptions about the procedure.

While MILs did recognize the benefits of using IUDs and pills, including spacing and improved child and maternal health before the next pregnancy, misconceptions around IUDs were common. Some MILs believed that IUDs may move around inside one’s body and cause injury. Stories of coerced IUD insertion at public facilities were also shared. Lastly, when asked about abortion, MILs were not supportive, and some described it as wrong and sinful.

#### FP Decision Making

Most MILs believed that whether to use FP and when to conceive should be a joint decision between husband and wife (Supplement 2).

*We should tell them that you both (son and DIL) have to discuss with each other. You should know each other. After discussion you can get the idea. We come to know about it when we discuss with him or her. People must communicate with each other. —*MIL, age 48 years, 10^th^ standard education

This highlights the importance of couples’ communication and wives’ consent and inclusion in decision making. A few MILs suggested that the DIL should be the final decision maker since it is her body and health that are most affected by childbearing.

*The pain which she suffers at the time of cesarean, only she can understand that. Men don’t have anything, so it is in women’s hands to take the decision about this whether she wants to have 1 child or more or whether they use family planning method or not. —*MIL, age 58 years, 7^th^ standard education

MILs felt that their opinion on the number of children should be considered but that their voice would not be heard or would be ignored by the couple because their son and DIL are more knowledgeable. Patrilocal norms were also reflected in some discussions with beliefs that the decision-making control should be with the MIL or son but that if the son made the decision it would take precedence. However, these norms are changing since some MILs think that either the DIL should make the decision or it should be made by the husband and wife together.

MILs reported they would value being involved in and participating in FP programs primarily for 2 reasons—to be part of couples’ decision making and to have an opportunity to learn (Supplement 2).

MILs reported they would value being involved in and participating in FP programs, both to be part of couples’ decision making and to have an opportunity to learn.

*Just like husband and wife, there should be participation of MILs and DILs. —*MIL, age 53 years, 10^th^ standard education

*Couple should discuss and sit like this [together]. We should also discuss about this [FP topics]. —*MIL, age 63 years, 4^th^ standard education

MILs wanted to participate in FP programs because they felt the programs may deliver information that would be good for them to learn. Furthermore, MILs believed they should be involved in programs since FP choices involve the family and intervention sessions may be conducted in homes and in the community.

*Everyone should participate in this program, because you are coming to our homes and giving such nice information. It sounds good. You are guiding us well. —*MIL, age 54 years, 10^th^ standard education

They suggested community or group discussions in addition to repeated couples’ counseling.

*You should conduct this type of activity (FGD activity) and also we should tell them properly. —*MIL, age 58 years, 8th standard education

*If you are visiting continuously and telling them then it will definitely beneficial. —*MIL, age 68 years, no education

## DISCUSSION

Our study is a qualitative exploration of the role MILs play in FP decision making and family life. Our findings suggest there is a preference among MILs to be included in FP decision making, early births in marriage, female sterilization over other modern forms of contraception such as pills and IUDs, and adherence to traditional gender roles for DILs in the home. These findings from rural Maharashtra are consistent with previous literature on MIL engagement in FP decision making from the northern Indian states of Bihar, Madhya Pradesh, and Uttar Pradesh,^9^^,^^18^^,^^21^ and extend this work by highlighting the interconnections of these types of preferences with traditional gender role expectations and early conception in marriage for their sons and DILs. At the same time, MILs acknowledged resistance to these traditional norms, recognizing that DILs often have more education and potential employment opportunities compared to women of their generation and thus need to alter norms to accommodate these generational shifts.

We found that some MILs were encouraging of their DILs’ ongoing education and income generation and the need for more shared household and childcare responsibilities. For these MILs, as well as for those MILs desiring more traditional norms, they anticipated their role as support for childcare, to allow DILs space for employment and/or domestic labor. This positioning as childcare support may be part of why many MILs feel entitled to participate in reproductive decision making with couples. Additionally, it should be noted that even among MILs supporting DILs’ continued education or employment, domestic labor responsibilities were still expected of the women but not their husbands. This places a double burden on DILs who choose to pursue education or livelihood opportunities because they still have to maintain responsibilities at home. The reinforcement of traditional gender roles such as domestic labor and caregiving on DILs goes hand in hand with MILs’ reinforcement of traditional fertility practices for their sons and DILs, including early-in-marriage conception and son preference. Given the apparent contradiction in expected and accepted DIL behavior by the MILs, further investigation is warranted into the challenges faced by DILs in straddling education, livelihoods, and agency over fertility versus the MILs’ expectations of domestic labor, early marriage and conception, and son preference.

The MILs in our study largely held negative attitudes toward modern contraceptives (preferring early births and subsequent sterilization) and this combined with pressures to conceive may affect contraceptive practices. We know from previous literature that MILs are able to influence birth outcomes within households. A study from Uttar Pradesh reported reproductive coercion by in-laws to be at a rate of 48%.^11^ Similar findings on the role of in-laws in engaging in pregnancy pressure tactics and related aspects of reproductive coercion have been reported in India, Ivory Coast, Jordan, Niger, and Kenya.^26^^–^^28^ Evidence also shows that women tend to turn to covert contraception use^29^^,^^30^ and female-controlled contraceptives^31^ in environments where their FP use is not supported. Even as they may feel ignored, MILs largely reported voicing their opinions to their sons and DILs, reported irritation when not heard, and reported a perceived right to influence decision making and voice their preferences. MIL influence is more likely as they expect entitlement to influence decision making. This entitlement to influence appears to be enforced by MILs’ role in childcare.

Our study found that MIL engagement in FP among couples is also linked to interest in participating in FP programming for couples. While this can be useful and important given the influence of MILs, it may inadvertently cost DILs control over their reproductive and contraceptive decision making. However, with findings from this study and recent literature that reiterates the role of MILs in FP, there is a clear need for FP programs to be sensitive to MIL attitudes and address related gender and fertility norms.

While including men in FP programs has been steadily gaining traction as a strategy to address gender-inequitable norms, much work needs to be done to understand the best ways to engage MILs in FP interventions. One way to incorporate our learnings into FP programming is to include a focus on gender equity in FP programming. Exploring and building on constructs of DILs’ FP agency—strengthening the capacity of DILs and/or couples to act on their FP goals given the pressures of MILs via intrahousehold communication, negotiation, contraceptive efficacy, digital access, and others—is emerging as a vital strategy. Previous literature has shown that social norms–based interventions can be effective in improving FP utilization.^32^^,^^33^ Community-based FP interventions that engage MILs as stakeholders may also hold promise. The Government of India is committed to this issue and implemented the Mission Parivar Vikas (MPV) FP program in 2016 in high-fertility districts in 7 states (not including Maharashtra). The MPV involves MILs through a MIL-DIL meeting activity called the Saas Bahu Sammelan to provide a platform for communication between pregnant and new mothers and their MILs.^20^

Improved understanding of MIL attitudes is needed to inform these initiatives. FP interventions need to address MILs’ attitudes and involvement in reproductive decision making while including a focus on gender equity and women’s agency, so as not to reinforce MILs’ control as decision makers. However, much more research and formative work need to be done to understand the best ways to engage MILs’ norms and attitudes in interventions in ways that do not detract from the DILs’/couples’ FP agency and goals. Given the large age range of 40 to 75 years for MILs, future research is also needed to understand whether there are segments of MILs, such as those who experienced early marriage themselves, that report different attitudes. Finally, triangulation of data between MILs and DILs may shed further light on the intergenerational disagreement and differences or similarities in fertility preferences that we found.

FP interventions need to address MILs’ attitudes and involvement in reproductive decision making while including a focus on gender equity and women’s agency, so as not to reinforce MILs’ control as decision makers.

### Limitations

Findings expand on the previously available knowledge on MILs in India and may be useful in similar intervention contexts. However, resulting from a qualitative study, our findings are not generalizable or representative. To be eligible for participation in this study, participants had to have their son and DIL enrolled in the CHARM2 intervention, so our findings are limited to this sample, and we do not have information on MILs whose sons and DILs did not enroll in CHARM2. Moreover, it is possible that these MILs may have been the ones to select DILs for their sons’ arranged marriage and their preferences already align. Another limitation is our convenience sampling method which only recruited the first 8–12 willing participants. This could introduce bias into participation. In the CHARM2 baseline sample, only 15.6% of wives reported being the primary decision maker on who to marry, and 9.4% reported that they had been the primary decision maker on when to marry.^34^ Previous estimates show that an overwhelming majority (94.3%) of marriages in rural Pune were arranged by parents and other relatives.^35^ MILs also may have approved their participation in CHARM2 since our findings show their desire to be involved in couples’ FP decisions. Since our study is limited to MILs’ perspectives and opinions, we are limited to their biases regarding their influence and engagement. Future research that includes DILs and sons should explore how MILs exert their influence on FP decision making and whether they are successful in doing so.

Our sample disproportionately consists of MILs from joint families, and so the findings may be more indicative of joint family situations where MILs have a stronger presence and more traditional practices. It should also be noted that we considered this MIL sample as one homogenous group, and further study on how age and education levels among MILs can influence FP perspectives and influences should be carried out. Also, this study site is from an agricultural rural area, where land ownership and inheritance likely affect views and practices related to fertility and son preference. The use of FGDs as a qualitative technique has some limitations in that although it elicits norms prevalent in society, in-depth interviews with individuals can better capture individual attitudes and behavior particularly when they are deviant from the norm. Although MILs largely reported beliefs that the couple should have joint decision making, some MILs said the son or MIL should make the decision. Individual interviews may be able to explore such differences in more detail. Our data were collected from August 2019 to February 2020, which was 9 months after the baseline survey and before the start of the intervention in September 2018. Thus, it is possible that some MILs may have experienced spillover effects of the CHARM2 intervention and reflected those in the opinions they shared. Finally, we conducted our FGDs before the lockdown and social-distancing measures implemented during the coronavirus disease (COVID-19) pandemic, so our study findings have not been distorted by this shock. Nevertheless, these public health measures may have impacted health services and extended family relationships in ways that are not captured here.

## CONCLUSIONS

Our study shows that MILs of young couples have traditional attitudes about motherhood, about their entitlement to be involved in their sons’ and DILs’ decision making, and about their DIL’s role in the family, all of which can compromise the centrality of DILs’ reproductive autonomy. Further, many also report a preference for childbearing early in marriage and large family size, nonuse of contraceptives outside of female sterilization, and son preference, which compromise the birth spacing options for women and couples. While some MILs have gender-equitable attitudes illustrated in their support for women’s education and income generation, they still maintain greater expectations of women’s versus men’s domestic labor for their DILs. Finally, MILs feel they have a role and responsibility to support the childcare for their sons and DILs, although this belief reinforces their entitlement to be involved with FP decision making. Future interventions may need to include components addressing MILs’ attitudes and involvement in FP decision making, not only because of their potential influence on the couple in perpetuating detrimental gender norms but also because they can compromise DILs’ reproductive autonomy.

Our study shows that MILs of young couples have attitudes about motherhood and their role in the couples’ reproductive decision-making process that can compromise the centrality of DILs’ reproductive autonomy.

## Supplementary Material

GHSP-D-22-00050-supplement.pdf

## Notes

**Peer Reviewed**

**First published online:** September 26, 2022.
