# Supplementary material for: Qualitative Examination of the Role and Influence of Mothers-in-Law on Young Married Couples’ Family Planning in Rural Maharashtra, India
Source: Glob Health Sci Pract. 2022 Oct 31;10(5):e2200050. doi: 10.9745/GHSP-D-22-00050 (PMC9622279; doi:10.9745/GHSP-D-22-00050)
Supplement: GHSP-D-22-00050-supplement.pdf [file GHSP-D-22-00050-supplement.pdf]

Supplement to: Dixit A, Ghule M, Rao N, et al. Qualitative examination of the role and influence of mothers-in-law on young married couples' family planning in rural Maharashtra, India. *Glob Health Sci Pract*. 2022;10(5):e2200050. https://doi.org/10.9745/GHSP-D-22-00050

SUPPLEMENT 1. Focus Groups Guide With Mothers-in-Law in Rural Maharashtra, India.

I am going to start by asking you a bit about how family dynamics work between the husband's parents and young couples.

1. Can you tell me a little about how the husbands' family is involved in the life of young married couples? (probe for mother-in-law roles in decision-making, helping daughter-in-law with domestic work and childcare)
2. What are the roles and responsibilities of the daughter-in-law in the family? What do mothers-in-law feel that their daughters-in-law should do in the family?
3. How are boys and girls brought up differently by their families to help them take on these types of responsibilities? Why?
4. What advice do mothers give their sons at the time of marriage? What do they say to help prepare them for married life?
5. Once married, how do mothers support their sons in their new families? How do they become involved if there are difficulties? (probe for abuse of wives/daughters-in-law)
6. What about in terms of family planning or deciding when to have children? How do parents discuss these issues with their young married sons? (probe for in-law interference, control)

Let's talk a bit more about these issues of family and family planning.

7. What are the family's attitudes towards the use of family planning methods? (probe around issues of early and high fertility, son preference, sterilization)
8. What do mothers/mothers-in-law know and understand about **modern** spacing methods of contraception?
9. What do mothers/mothers-in-law think about these modern contraceptive methods? What are the perceived advantages and disadvantages?
10. Who are the family members who exert influence over a young couple's decisions about family planning? What is their position and role within the family? To what extent do they influence the family planning decisions?
11. Who do you think should make decisions regarding the use of family planning methods? Why?
12. What changes need to occur within a family to make the family more receptive to the use of modern family planning methods?
13. How can men's (sons') attitudes be changed to be more receptive to family planning and family planning methods?
14. How can in-laws support this? (probe for how in-laws may be involved with a clinic-based family planning program)

As you know, our CHARM2 program is a family planning program for young married couples in this village. I want to describe this program to you and get your feedback on it and how you think it will help your family and community.

- *The CHARM2 program offers family planning counseling from a male provider for husbands and from a female provider for wives, to help the couple build their family planning knowledge and support for marital relationship issues. A final session will be given to the couple to offer them family planning services and marital communication counseling. All sessions and services will be given for free.*

15. What are your thoughts on the program (probe for how in-laws support or engage with the program)?

16. How would you like to see the community and other family members involved in this program? (Probe for how in-laws should or should not be included, how peer support could be useful, what community awareness efforts could help- street theatre)

17. Are there any other ideas or comments you have for the CHARM2 program?

<u>Closure</u>. *Thank you so much for your participation. Your answers will help us make the best possible program for families in this community..*
**Following focus group completion, participants will again be given referrals to the ASHA and Primary Health Center.

Supplement to: Dixit A, Ghule M, Rao N, et al. Qualitative examination of the role and influence of mothers-in-law on young married couples' family planning in rural Maharashtra, India. *Glob Health Sci Pract*. 2022;10(5):e2200050. https://doi.org/10.9745/GHSP-D-22-00050

SUPPLEMENT 2. Thematic Analysis and Quotes From Focus Groups With Mothers-in-Law in Rural Maharashtra, India (N=86 Participants, 10 FGDs)

Theme	Sub-theme	Quote
Mother-in-law role in family life	Parent and guide for daughter-in-law	"I: How did you get involved with the couple in your family?<br>R 4: We should love our daughter-in-law. We should leave some things up to them. We should not treat them as a daughter-in-law. We should treat them as a daughter.<br>R 5: They make mistakes just like our daughter, so we should understand them accordingly." (FGD 1; R5 age 60, education 3rd standard)<br><br>"R 6: Now, if we say a single word then. [conflict will occur]<br>R 1: They will immediately run away (all were laugh) and say that we are going to live separately. This can continue till they say that mother-in-law is a stranger." (FGD 4; R1 age 52 years, education 7th standard; R6 age 50 years, education 5th standard)<br><br>"I: How do you guide her if she has made any mistake?<br>R 6: Nowadays nobody has to say these things. We were minor [age 18] at the time we got married. Now these girls get married after they are adult, so they have knowledge of everything. If we tell them anything, they get angry.<br>R 6: So telling them anything is wrong. We never brought illiterate girls [home after marriage]. They are well educated. When we got married at that time, we had a lot of responsibilities, but now these girls don't have that much responsibility." (FGD 10; R6 age 58, education 7th standard)
	Parent and protector of son	"I: What is your role as a mother-in-law in the marital life of your son and daughter in law?<br>R-5: Our role is to love our son. Being a mother-in-law our role is to check whether their marital life is going well or not. The marital life of our son should be proper. This is an expectation we have. We do not have further expectation. We do not want to do anything if he is happy in his marital life. " (FGD 2; R5 age 48, education 4th standard)
	Caregiver for grandchildren	"I: What is your opinion about taking care of children? Who should take care of that?<br>R 1: Grandmother has the responsibility to take care of baby.<br>R 2: Yes, this is the responsibility of grandmother.<br>R 3: But if she (daughter-in-law) will allow children to come to us then only we can take care of them. She has to understand that grandmothers have some responsibility. She should know this." (FGD 7; R1 age 50, education 9th standard; R2 age 54, education none; R3 age 59, education 2nd standard)<br><br>"R 1: We are taking care of their children. We also trying to make their family happy. If they have some issues then we help them to sort those out. They help us and we also help them. This is what we expect." (FGD 3; R1 age 55, education none)
Expected role of daughter-in-law	Domestic labor for the family	"I: What responsibilities should your daughter-in-law carry out in your family?<br>R 1: She has to cook food twice a day. She has to wash clothes and utensils. She has to take care of grandchildren. She has to check on the in-laws and we expect nothing more than that." (FGD 8; R1 age 45, education 6th standard)<br><br>"I-1: Which responsibilities do they have?<br>R-4: She cooks and does all the necessary things. To make food and give tiffin to children as well as to look after them as they are kids. They do lots of domestic work. Whole day passes while looking after the children. We are really grateful to see this." (FGD 2; R4 age 59, education 7th standard)<br><br>"I: What are your expectations now, tell me?<br>R 4: Our only expectation is they should do the things as we have done (e.g., domestic labor, care for family including in-laws), nothing different than that.<br>R 3: Whatever we have done, they should also do the same things in future." (FGD 1, R3 age 61, education 8th standard)
	Educated and income generating	"I: What things do they have to learn?

Supplement to: Dixit A, Ghule M, Rao N, et al. Qualitative examination of the role and influence of mothers-in-law on young married couples' family planning in rural Maharashtra, India. *Glob Health Sci Pract*. 2022;10(5):e2200050. https://doi.org/10.9745/GHSP-D-22-00050

Theme	Sub-theme	Quote
		R 1: They should not spend their time how we have spent in those days, under our mother-in-law. I think my daughter-in-law has to go out. She has to know the world. She should be knowledgeable on financial matters. I expect all these things." (FGD 10, R1 age 48, education 10th standard)<br><br>"I: Tell me your opinion about when they should have children?<br>R 7: You have grandson, right? (to another respondent)<br>R 3: Yes, I am taking care of him. My only expectation is, I want one more child. I will take care of that kid. She [my daughter in law] is not going for job also. She got calls for job. She has completed her graduation and she is a ranker (got high marks) from 12th class. I told her to get further education. Here she needs to work at home so I sent her to her parents home." (FGD 5; R7 age 48, education 4th standard; R3 age 53, education 2nd standard)<br><br>"I 1: According to you what is important? Job or household chores?<br>R3: They should manage their time for family also. They should take care of their children's future. Whatever we have done for our children, they should do that for their children.<br>R 5: We never expect everything from daughter-in-law but she should manage her time for her family and her children, we never expect that she should earn more money but she should be careful all the time." (FGD 3; R3 age 45, education 7th standard; R5 age 60, education 4th standard)
Norms and attitudes toward family planning	Fertility norms and attitudes: Early in marriage fertility	"I: What is your opinion about when they should have children?<br>R 4: They should have children immediately (after marriage). They should have a first child in 2 to 3 years of their marriage, whether it is a son or daughter.<br>R 6: After completing 1 year of their marriage everybody expects a child from them.<br>R 1: Even though we expect, they should also think about it." (FGD 1; R4 age 58, education 8th standard; R6 age 68, education none; R1 sociodemographics missing)<br><br>"R 5: It gets more harmful if we have child so late. As the age increases, they will have more problems. We should have everything in time and in proper age.<br>R 4: Now we are capable to take care of grandchildren." (FGD 1, R4 age 58, education 8th standard)<br><br>"R 9: Yes, if they take contraceptive pills immediately after marriage they get problems.<br>R 1: They can use it after having a child but they should not use it before having a child." (FGD 7; R9 age 40, education none; R1 age 50, education 9th standard;)<br><br>"I : Do you think that they should not use that [a contraceptive method] before having first child?<br>R 6: They should not do anything before having first child, later they can use whatever they want.<br>R 10: If they use anything [a contraceptive method] they can't conceive when they want to have the baby." (FGD 10; R6 age 58, education 7th standard; R10 age 60, education 5th standard)
	Fertility norms and attitudes: Son preference	"R 4: Yesterday I told my son that we should have at least one son. My older son has two daughters and younger son has one daughter, so I tell them that we should have a son. So my younger son tells me that he will live happily after his daughter's marriage. They both (older and younger son) are satisfied with only daughters." (FGD 1, R4 age 58, education 8th standard)<br><br>"I: Now tell me what is your opinion about having son or daughter?<br>R 3: Tell everybody the truth. Now we have that capacity to tolerate. My daughter has done the operation (sterilization) after having 2 daughters. But sometimes we feel that she should have 1 son after having 2 daughters. We think that son is superior.<br>R 3: Yes, we feel that. Now we say that we don't discriminate, but that is not true, everybody wants a son. (FGD 7; R3 age 59, education 2nd standard)<br><br>"R 3: Yes, daughter can be an inheritor but actually these are all formalities. Even if son gives us pain or he gives us any trouble, still people can tolerate that. If we live in our village, we can live proudly but we can't live in our daughter's home with pride. We easily say that we can stay with daughters but it is not true in reality." (FGD 7; R3 age 59, education 2nd standard)<br><br>"I-1: There are some families who have son preference. Do you have such preferences in your family?

Supplement to: Dixit A, Ghule M, Rao N, et al. Qualitative examination of the role and influence of mothers-in-law on young married couples' family planning in rural Maharashtra, India. *Glob Health Sci Pract*. 2022;10(5):e2200050. https://doi.org/10.9745/GHSP-D-22-00050

**Theme**	**Sub-theme**	**Quote**
		R-4: Here you can see daughters are more educated than the sons. Boys have ancestral land but girls take education without thinking of the ancestral land. Hence all girls are getting educated.<br>R-10: In our Vdagaon Anand, girls are well educated. Girls understand everything and not boys.<br>R-10: It is not there but earlier it was there." (FGD 2, R4 59 years education 7th standard; R10 age 54 years, education 10th standard)
	Fertility norms and attitudes: Higher fertility than couple desires	"I-1: But ever this happens that you want two children in the family, and they have decided to have one child?<br>R-5: In old days, population was increasing but not now, no one is ready to increase population. No one is ready to give birth above two children.<br>R-10: No one is ready for third child. Two children are ok. Some says that one child is enough." (FGD 2; R5 age 48, education 4th standard; R10 age 54, education 10th standard)<br><br>"I 1: What do you think, do you want one more?<br>R 1: We want 10 grandchildren (laughs) but they are saying that they want only one.<br>R 3: So we never advise them anything. They will do whatever they want to do.<br>R 4: Suppose, we tell them to have a second child and if they never carry out the responsibility of one child then what will happen? They will say that you had expected a second child. So what we will answer them?" (FGD 3; R1 age 55 education none; R3 age 45, education 7th standard; R4 age 55, education 7th standard)
	Birth spacing norms and attitudes	"I: What do you think; when should they have the children or how many children should they have?<br>R 3: I think they should keep the spacing of 4 to 5 years and we also expect that it takes at least 2 years to have the baby.<br>R 4: I also feel that they should keep 2 to 4 years spacing. I think she should have the child after 4 to 5 years." (FGD 10; R3 age 45, education none; R4 age 55, 1st standard)<br><br>"I: What do you think; should they have a second baby after some years of space?<br>R 10: Yes, we feel that they should keep space between two children. We think that if they have daughter, they should have son after 4 to 5 years. They should have first baby after 2 years of their marriage. (FGD 9; R10 age 60, education 5th standard)
	Contraceptive norms and attitudes: Pill	"I: As you mentioned about contraceptive pills; now tell me is taking contraceptive pills right or wrong?<br>R 9: No, taking contraceptive pills is not right. Yes, it is not right, we want a child, however it is.<br>R 3: They can do operation immediately after that. (FGD 7; R9 age 40, education none; R3 age 59, education 2nd standard)<br><br>"I-1: What do you think about the pills? What is your opinion about pills?<br>R-4: Pills are not appropriate to consume.<br>R-11: It is wrong to consume the pills. Due to pills, they can miss periods. It's my personal experience. During our days so many women were consuming pills but no effect was found.<br>R-3: Pills are not good for the health<br>. If pregnancy happens let it happen. That will give company to previous child." (FGD 2; R4 age 59, education 7th standard; R11 age 50 education 8th standard; R3 age 49 education 7th standard)
	Contraceptive norms and attitudes: Male sterilization	"I 2: Suppose you have a married son and he says that he is planning to go for male sterilization, what will be your opinion?<br>R 4: No, that creates a problem.<br>R 4: Men have heavy workload so he will say that he will not be ready to do that.<br>R 6: Men do not do that.<br>I: No, not for men?<br>R 4: Because men have heavy work so…<br>R 6: Women don't have heavy work." (FGD 1; R4 age 58, education 8th standard; R6 age 68, education none)<br><br>"I-1: As you said males have to take more care, so can you tell me who needs to do the operation? I mean male sterilization or female sterilization?<br>R-5: Daughter in law.<br>R-4: Usually daughter in laws.

Supplement to: Dixit A, Ghule M, Rao N, et al. Qualitative examination of the role and influence of mothers-in-law on young married couples' family planning in rural Maharashtra, India. *Glob Health Sci Pract*. 2022;10(5):e2200050. https://doi.org/10.9745/GHSP-D-22-00050

Theme	Sub-theme	Quote
		R-2: Son has to do. What is there if son undergoes. 99% women does operation so at least 1% male has to do operation.<br>R-4: Women have nothing to do. What is the problem with male sterilization?<br>R-5: Male has to work outside and women have to work inside the house." (FGD 2; R5 age 48, education 4th standard: R4 age 59, education 7th standard; R2 age 53, education 10th standard)<br><br>"I 1: What do you think? Who should do this, female or male?<br>R 2: It should do only female, because men have enough hard work than women.<br>I 1: But why women should do that?<br>R 3: Men have to carry heavy objects and women not.<br>R 2: This is the tradition going on from past.<br>R 3: Men can earn Rupees 500 per day, but women earn only 200 per day so anything happens with female, men can handle the financial situation and he can carry out his responsibilities through his income. But woman can't manage with her rupees 200."<br>(FGD 3; R2 age 65, education 5th standard; R3 age 45, education 7th standard)
	Contraceptive norms and attitudes: IUD	"I: Do you know the advantages and disadvantages of these methods?<br>R 6: My daughter-in-law had inserted the Copper T (IUD) but she was looking so thin after inserting it so she removed it last year. She suffered a lot due to it.<br>R 5: Yes, it is not suitable for everybody.<br>R 4: Yes, it never suits everybody.<br>R 6: Sometimes some women have vomiting." (FGD 1, R6 age 68, education none; R4 age 58, education 8th standard; R5 age 60, education 3rd standard)<br><br>"I 2: Is there any advantages or disadvantages of that?<br>R 10: It is good. Injections are better than Copper T (IUD).<br>I 2: Why is it like this?<br>R 10: Copper T may hurt, it may also move ahead in body. We don't know about it, but girls tell us and some get injured through it.<br>R 8: If we take medicines (pills), we can have heavy bleeding." (FGD 9; R10 age 60, education 5th standard; R8 age 50, education 4th standard)<br><br>"I 1: What about Copper T?<br>R 2: Some methods can be annoying; we have experience about Copper T, doctors in government hospital has forced us to insert that." (FGD 3; R2 age 65, education 5th standard)<br><br>"I: Are tablets and Copper T good?<br>R 4: No, it is not good.<br>I 2: Did your daughter-in-law get complications?<br>R 4: Yes, she got some complications and she looked thin due to them. When that madam visited, she inquired about her health?<br>I 2: Did she visit doctor?<br>R 4: Then she went and she removed Copper T.<br>I 2: Ok.<br>R 4: There was pain only in her lower abdomen. She told me then I told her to remove it. I heard that from outsiders also. Then she went to her parents' house and she removed it in a government hospital.<br>I: Where did she inserted it?<br>R 4: In a government. hospital where her delivery took place. We were not aware of it. When she came here she was looking so thin. I just asked her, why are you looking like this? She told me that she has pain in her lower abdomen due to Copper T. I asked her, who has given you the permission for insertion. She said that there is rule in government hospital to insert Copper T after delivery. I just refused it and I said there is no rule anywhere like this. I think she got scared so she told me a lie." (FGD 1, R4 age 58, education 8th standard)<br><br>"I: Do you know the advantages and disadvantages of the Copper T and tablets?

Supplement to: Dixit A, Ghule M, Rao N, et al. Qualitative examination of the role and influence of mothers-in-law on young married couples' family planning in rural Maharashtra, India. *Glob Health Sci Pract*. 2022;10(5):e2200050. https://doi.org/10.9745/GHSP-D-22-00050

Theme	Sub-theme	Quote
		R 6: If we insert the Copper T for spacing it is better for a child, He or she can grow better, and women also have some gap to cover her health issues because she doesn't have that stamina to give birth to another child." (FGD 9; R6 age 48, education 10th standard)
	Contraceptive norms and attitudes: Abortion	"I: Now tell me, what is your opinion about abortion?<br>R 4: One should not do that.<br>I: What do you think? Is it right or wrong?<br>R 6: No. It is wrong.<br>R 4: It is wrong.<br>R 1: Nowadays we can see that sometimes somebody can't conceive a pregnancy and whoever can conceive they abort it.<br>R 4: It is sin." (FGD 10; R4 age 55, 1st standard; R6 age 58, education 7th standard; R1 age 48, education 10th standard)
Family planning decision-making	Couple or female decision-making on fertility goals and contraceptive use	"I: So whose responsibility it is?<br>R-10: Both of them have this responsibility.<br>R-4: Both can decide not to give birth to a child, if they think. And they have to do operation.<br>R-2: If they do not want a child, then they have to do operation. And if they want a child then they can decide which method can be used." (FGD 2; R10 age 54, education 10th standard; R4 age 59, education 7th standard; R2 age 53, education 10th standard)<br><br>"R 1: We should tell them that you both (son and daughter-in-law) have to discuss with each other. You should know each other. After discussion you can get the idea. We come to know about it when we discuss with him or her. People must communicate with each other.<br>R 2: I told my son that she (daughter-in-law) comes from outside so we have to take care of her. If she makes any mistake, we have to tell her properly." (FGD 10; R1 age 48, education 10th standard; R2 age 56, education 10nd standard)<br><br>"I: Who takes decision about family planning methods in your family?<br>R 2: I think the couple should take the decision.<br>R 8: Woman should do this.<br>R 6 & 8: Women should take it together (with husband).<br>R 1: I think it should be taken with the consent of both.<br>R 5: She (daughter-in-law) should listen to us and we should also listen to her." (FGD 4; R2 age 62, education non; R8 age 42, education 10th standard; R6 age 50, education 5th standard; R1 age 52, education 7th standard; R5 age 48, education 5th standard)<br><br>"I: What do you think in these couples? Who should take the decision of using family planning methods? Son or daughter-in-law?<br>R 2. 4. 5. 6: They both should take the decision.<br>R 6: Daughter-in-law should take the final decision.<br>R 2: Daughter-in-law should take the decision, because she tolerates that pain.<br>R 6: If any woman has pregnancy, either she can have normal delivery or she may have cesarean. And the pain which she suffers at the time of cesarean, only she can understand that. Men don't have anything, so it is in women's hands to take the decision about this whether she wants to have one child or more or whether they use family planning method or not." (FGD 10; R2 age 56, education 10th standard; R6 age 58, education 7th standard)
	Mother-in-law engagement in decision-making: Should be considered but often ignored	"I: Do you think that there is a need to take your opinion about how many children they want, or decision about their family planning?<br>R 3: Yes, should take our opinion in that.<br>R 1: Yes, it should be, but now who listens?<br>R 2: They never listen.<br>R 5: But we want that they should listen to us.<br>R 1: They never listen.<br>R 1: Now I have told all 3 of my sons, that nobody will do operation (sterilization). Also I have told my daughters-in-law." (FGD 5; R3 age 53, education 2nd standard; R1 age 52, education none; R2 age 63, education 4th standard; R5 age 48, education 4th standard)

Supplement to: Dixit A, Ghule M, Rao N, et al. Qualitative examination of the role and influence of mothers-in-law on young married couples' family planning in rural Maharashtra, India. *Glob Health Sci Pract*. 2022;10(5):e2200050. https://doi.org/10.9745/GHSP-D-22-00050

Theme	Sub-theme	Quote
		R11: Nowadays nobody asks others. They do it on their own. They take the tablets. They don't even ask. Really, they are not bothered to tell us the fact. Why should we bother?<br>R5: They should understand that our mother-in-law is like our mother. She should understand that she shouldn't have taken any decision without telling family. We should not hold the control over them. We can't hold their hand like this." (FGD 6; R11 age 65, education non; R5 age 65, education 9th standard)<br><br>R 5: In my family, I can take the decision about this (use of birth control). But if they don't want any more children, so what can we do for that? So we also keep quiet." (FGD 4; R5 age 48, education 5th standard)<br><br>"I: Now tell us who should decide the family planning methods which the couple want to use?<br>R 10: Mother-in-law or son but if son has taken the decision there is no need of mother.<br>R 1: Mother-in-law or son should take the decision.<br>R 6,10: Daughter-in-law can also take that decision.<br>R 6: I think they both (husband and wife) should take this decision.<br>R 11: They can take their decision, but we should also tell them." (FGD 9; R10 age 60, education 5th standard; R1 age 55, education 4th standard; R6 age 48, education 10th standard)
	MIL engagement in decision-making: Unnecessary	"I: They should do the things according to your opinion?<br>R 4: They all are young. They understand everything. Why should we expect from them?<br>R 4: We did not understand these things before. Nowadays these children know about these things. We do not need to tell them anything." (FGD 1; R4 age 58, education 8th standard)
	Desire to be involved in and learn from family planning programs	"I: Which information is needed to give?<br>R 1: We should also know the things.<br>R 3: Yes. You should also give this information to mother-in-law." (FGD 8; R1 age 45, education 6th standard; R3 age 65 education 7th standard)<br><br>"I: Whose participation is needed to increase? What do you think?<br>R-10: Everyone should participate in this program, because you are coming to our homes and giving such nice information. It sounds good. You are guiding us well.<br>R-2: Daughters in law will get more information and they will understand the program very well. We are speaking four words but as you are coming to our homes and discussing with us, it sounds good. They usually share so and so information I got today, or today I got very different information." (FGD 2; R10 age 54, education 10th standard; R2 age 53, education 10th standard)<br><br>"R4: You should conduct this type of activity (FGD activity) and also we should tell them properly<br>R 6: If you are visiting continuously and telling them than it will definitely beneficial.<br>R 4: If our family is happy then everybody stays happy and it is beneficial for them. If they can understand everything, they can plan according to that. Taking education is become expensive now so I think this program is really good. Government is really conducting nice activity through this." (FGD 1, R4 age 58, education 8th standard, R6 age 58, education 7th standard)

Abbreviation: FGD, focus group discussion; IUD, intrauterine device; R, respondent.
